# Supplementary material for: P-TRAP: a Panicle Trait Phenotyping tool
Source: BMC Plant Biol. 2013 Aug 29;13:122. doi: 10.1186/1471-2229-13-122 (PMC3848748; doi:10.1186/1471-2229-13-122)
Supplement: Additional file 7 — P-TRAP source code. The Netbeans project that contains the source code of the application. [file 1471-2229-13-122-S7.zip › javadoc.zip!/javadoc/org-bioalg-riceprojecttype/org/bioalg/riceprojecttype/RiceProjectFactory.html]

RiceProjectFactory (RiceProjectType)


JavaScript is disabled on your browser.


- Package
- Class
- Tree
- Deprecated
- Index
- Help

*org.bioalg.riceprojecttype 1.0*

- Prev Class
- Next Class

- Frames
- No Frames

- All Classes

- Summary:
- Nested |
- Field |
- Constr |
- Method

- Detail:
- Field |
- Constr |
- Method


org.bioalg.riceprojecttype

## Class RiceProjectFactory

- java.lang.Object
- - org.bioalg.riceprojecttype.RiceProjectFactory

- All Implemented Interfaces:
  :   org.netbeans.spi.project.ProjectFactory

  ---

    

  ```
  public class RiceProjectFactory
  extends java.lang.Object
  implements org.netbeans.spi.project.ProjectFactory
  ```

  The projects creator class.

  Author:
  :   faroq

- - ### Field Summary

    Fields

    | Modifier and Type | Field and Description |
    | --- | --- |
    | `static java.lang.String` | `IMAGE_DIR` |
    | `static java.lang.String` | `PARTICLES_RESULT_DIR` |
    | `static java.lang.String` | `PARTICLES_RESULT_FILE` |
    | `static java.lang.String` | `PROJECT_DIR` |
    | `static java.lang.String` | `RESULTS_DETAILS_FILE` |
    | `static java.lang.String` | `RESULTS_DIR` |
    | `static java.lang.String` | `RESULTS_MASTER_FILE` |
  - ### Constructor Summary

    Constructors

    Constructor and Description

    `RiceProjectFactory()`
  - ### Method Summary

    Methods

    | Modifier and Type | Method and Description |
    | --- | --- |
    | `boolean` | `isProject(org.openide.filesystems.FileObject projectDirectory)` |
    | `RiceProject` | `loadProject(org.openide.filesystems.FileObject dir, org.netbeans.spi.project.ProjectState state)` |
    | `void` | `saveProject(org.netbeans.api.project.Project project)` |

    - ### Methods inherited from class java.lang.Object

      `clone, equals, finalize, getClass, hashCode, notify, notifyAll, toString, wait, wait, wait`

- - ### Field Detail


    - #### PROJECT\_DIR

      ```
      public static final java.lang.String PROJECT_DIR
      ```

      See Also:
      :   Constant Field Values


    - #### IMAGE\_DIR

      ```
      public static final java.lang.String IMAGE_DIR
      ```

      See Also:
      :   Constant Field Values


    - #### RESULTS\_DIR

      ```
      public static final java.lang.String RESULTS_DIR
      ```

      See Also:
      :   Constant Field Values


    - #### RESULTS\_MASTER\_FILE

      ```
      public static final java.lang.String RESULTS_MASTER_FILE
      ```

      See Also:
      :   Constant Field Values


    - #### RESULTS\_DETAILS\_FILE

      ```
      public static final java.lang.String RESULTS_DETAILS_FILE
      ```

      See Also:
      :   Constant Field Values


    - #### PARTICLES\_RESULT\_DIR

      ```
      public static final java.lang.String PARTICLES_RESULT_DIR
      ```

      See Also:
      :   Constant Field Values


    - #### PARTICLES\_RESULT\_FILE

      ```
      public static final java.lang.String PARTICLES_RESULT_FILE
      ```

      See Also:
      :   Constant Field Values
  - ### Constructor Detail


    - #### RiceProjectFactory

      ```
      public RiceProjectFactory()
      ```
  - ### Method Detail


    - #### isProject

      ```
      public boolean isProject(org.openide.filesystems.FileObject projectDirectory)
      ```

      **Specified by:**
      :   `isProject` in interface `org.netbeans.spi.project.ProjectFactory`


    - #### loadProject

      ```
      public RiceProject loadProject(org.openide.filesystems.FileObject dir,
                            org.netbeans.spi.project.ProjectState state)
                              throws java.io.IOException
      ```

      **Specified by:**
      :   `loadProject` in interface `org.netbeans.spi.project.ProjectFactory`

      Throws:
      :   `java.io.IOException`


    - #### saveProject

      ```
      public void saveProject(org.netbeans.api.project.Project project)
                       throws java.io.IOException,
                              java.lang.ClassCastException
      ```

      **Specified by:**
      :   `saveProject` in interface `org.netbeans.spi.project.ProjectFactory`

      Throws:
      :   `java.io.IOException`
      :   `java.lang.ClassCastException`


- Package
- Class
- Tree
- Deprecated
- Index
- Help

*org.bioalg.riceprojecttype 1.0*

- Prev Class
- Next Class

- Frames
- No Frames

- All Classes

- Summary:
- Nested |
- Field |
- Constr |
- Method

- Detail:
- Field |
- Constr |
- Method
